# On the Question of the Application Potential and the Molecular Mechanism of the Formation of 1,3-Diaryl-5-Nitropyrazoles from Trichloromethylated Diarylnitropyrazolines

**DOI:** 10.3390/molecules30214306

**Published:** 2025-11-05

**Authors:** Karolina Kula, Radomir Jasiński

**Affiliations:** Cracow University of Technology, Faculty of Chemical Engineering and Technology, Department of Organic Chemistry and Technology, Warszawska 24, 31-155 Cracow, Poland

**Keywords:** nitrocompounds, elimination reaction, molecular mechanism, molecular electron density theory, density functional theory, activity distribution metabolism and excretion, prediction of activity spectra for substances

## Abstract

The molecular mechanism of the formation of 1,3-diaryl-5-nitropyrazoles via a CHCl_3_-elimination reaction was investigated using ωB97xD/6-31+G(d,p) (PCM) calculations. It was found that, regardless of the polarity of the reaction environment or the nature of the substituents on the phenyl rings of the starting molecules, the elimination process proceeds through a single-step mechanism characterized by an extremely asynchronous transition state. The ELF (Electron Localization Function) analysis of selected critical structures confirms the proposed mechanism and reveals a pronounced reorganization of electrons within the heterocyclic ring. The in silico analysis based on ADME (Activity, Distribution, Metabolism, and Excretion) and PASS (Prediction of Activity Spectra for Substances) predictions indicates that the title 1,3-diaryl-5-nitropyrazoles exhibit promising biological potential, showing inhibitory activity against both oxidoreductases and proteases. The most consistent targets include hyponitrite reductase, (*R*)-6-hydroxynicotine oxidase, acrocylindropepsin, saccharopepsin, and chymosin. Thus, the presented CHCl_3_-elimination provides an efficient and versatile route to functionalized pyrazoles, and, together with their promising bioactivity, confirms the utility of this approach for their synthesis.

## 1. Introduction

The pyrazole heterocyclic skeleton is a well-recognized structural motif that plays a central role in the architecture of numerous bioactive compounds [1,2]. It is found in several clinically relevant pharmaceuticals, including *Celecoxib*, *Tepoxalin*, *Anagliptin* or *Crizotinib*, which underlines its importance in modern medicinal chemistry [3,4,5]. A particularly interesting modification of this scaffold involves the introduction of a nitro substituent. The nitro group has been shown to provide additional stimulation of biological activity [6,7], thereby enhancing the pharmacological potential of various heterocycles, including pyrazole derivatives [8,9]. Furthermore, nitro-substituted heterocycles have been highlighted as promising candidates for drug discovery in several independent studies [10,11]. Beyond their intrinsic bioactivity, nitro groups serve as versatile functional handles, enabling a broad spectrum of subsequent derivatizations [12,13,14]. This synthetic flexibility significantly expands the chemical space available for structure–activity relationship (SAR) studies [15,16]. The opportunities for further modification of nitropyrazoles have been explored extensively in recent years. For instance, new strategies for selective derivatization of nitroaromatic systems have been reported, illustrating their synthetic versatility [17]. Complementary approaches have revealed new pathways to functionalized heterocycles [18], while earlier studies established the classical reactivity of nitropyrazoles [19,20,21], highlighting the biological and synthetic appeal of these analogs.

**Scheme 1:**
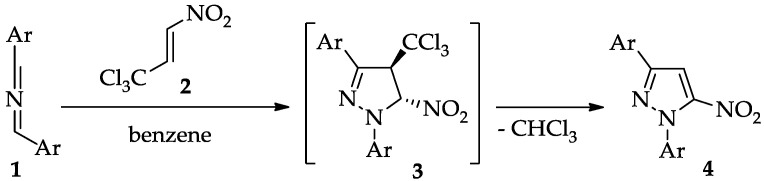
The formation of 1,3-diaryl-5-nitropyrazoles (**4**) via domino reaction based on sterically nitrilimines (**1**) and the trichloronitropropene (**2**).

From a methodological standpoint, the most universal and selective protocol for constructing the pyrazole framework is the (3 + 2) cycloaddition (32CA) reaction. Traditionally, this process involves alkynes and diazo compounds, or alternatively nitrilimines [22,23,24], acting as three-atom components (TACs) [25]. This strategy has been widely applied owing to its high efficiency and selectivity, and it remains the cornerstone of pyrazole synthesis [26]. Despite these advantages, the classical 32CA route is not applicable for the preparation of nitro-functionalized pyrazoles, since nitroacetylenes are unstable and cannot be used as practical synthetic reagents [27,28]. To overcome this limitation, alternative methodologies have been proposed. One particularly attractive approach relies on 32CA reactions of conjugated nitroalkenes, followed by the aromatization of the initially formed pyrazoline intermediate [29,30]. In line with this concept, we recently developed a convenient route toward 1,3-diaryl-5-nitropyrazoles (**4**). In this methodology, the primary cycloadduct (**3**) formed in the benzene solution was found to be stable only at low temperatures (about 5–10 °C). The lifetime of the molecular individuum is not long, but it was sufficient to perform the analysis. Upon mild heating (about 30–35 °C), however, it spontaneously underwent conversion into the target nitropyrazole (**4**) via the elimination of a chloroform molecule (Scheme 1) [31]. This mechanistic insight not only broadens the scope of nitropyrazole synthesis but also sheds light on the reactivity of pyrazoline intermediates, paving the way for future exploration of this chemistry.

The mechanistic aspects of these syntheses have so far been investigated only with respect to the initial cycloaddition stage. In contrast, the subsequent conversion of nitropyrazolines (**3**) into nitropyrazoles (**4**) has not been analyzed in any systematic way. The present study represents a continuation of our earlier research [31] and specifically addresses the molecular mechanism of the elimination processes occurring in nitropyrazoline systems [32,33]. In this context, we have identified several key issues that require clarification, including the following:

(i) *The molecular mechanism of chloroform elimination*

The first question concerns the exact nature of the chloroform elimination step. Theoretically, three different mechanistic scenarios can be envisaged for the elimination of small molecules from this type of heterocyclic intermediate. The first possibility is a single-step pseudocyclic elimination, a pathway previously reported for related systems [34,35,36]. The second option is a stepwise elimination following an E1-type pathway, which involves the initial departure of a leaving group with subsequent carbocation formation [37,38]. Finally, a third scenario is the E1cb-type pathway, characterized by initial deprotonation to generate a carbanion, followed by elimination of the leaving group [39,40]. Distinguishing between these alternatives is crucial for a reliable mechanistic picture.

(ii) *The generality of the elimination mechanism and the role of substituents*

Another important issue is whether the mechanism of chloroform elimination can be considered general across a broader range of structurally related compounds, or whether it is highly substrate-specific. In this context, the substituent effect becomes a central factor. Electron-donating or electron-withdrawing groups attached to the aryl rings of nitropyrazolines may strongly influence the stability of possible intermediates, the synchronicity of bond-breaking processes, and the overall energetic profile of the elimination step. Understanding how these substituents alter the mechanistic pathway will provide deeper insight into the scope and limitations of this transformation.

(iii) *The influence of solvent polarity*

The polarity of the reaction medium is also expected to play a crucial role in determining the course of the elimination. It is therefore necessary to address whether the reaction mechanism is invariant to solvent polarity or, alternatively, whether different mechanisms can be enforced depending on the dielectric properties of the medium. For instance, a polar solvent may stabilize ionic intermediates, favoring stepwise E1 or E1cb-type pathways, whereas in less polar environments a single-step pseudocyclic mechanism may be preferred. Clarifying the role of solvent effects will not only rationalize experimental outcomes but also offer practical guidelines for tuning the mechanism by deliberate choice of reaction conditions.

(iv) *The electronic nature of the transition states*

Finally, the precise electronic nature of the transition states (TSs) involved in the elimination must be elucidated. The degree of asynchronicity in bond-breaking and bond-forming processes, the extent of charge separation, and the stabilization of developing electronic structures are all key descriptors of the TS character. These features can provide a unified picture linking structural, electronic, and environmental effects to the observed reactivity.

**Scheme 2:**
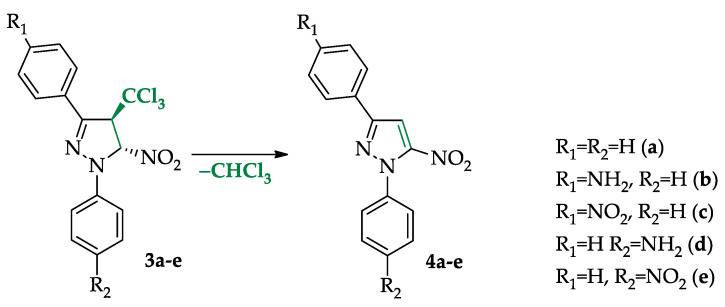
Model reactions analyzed in the framework of the research.

To address these mechanistic questions, we employed the framework of the Molecular Electron Density Theory (MEDT) [41], based on Density Functional Theory (DFT) [42] calculations. This approach has recently been successfully applied to interpret the mechanisms of a wide variety of organic transformations [43,44,45,46]. Within this methodological framework, a homogeneous series of model reaction components was selected (Scheme 2), ensuring a consistent analysis of substituent and solvent effects as well as electronic features of the relevant transition states.

Additionally, we performed preliminary in silico analyses to evaluate the potential biological activity of the target 1,3-diaryl-5-nitropyrazoles (**4a**–**e**). These studies employed both ADME (Activity, Distribution, Metabolism, and Excretion) [47] and PASS (Prediction of Activity Spectra for Substances) [48] predictions to assess pharmacokinetic properties, drug-likeness, and possible bioactivity profiles. The results provide initial evidence supporting the promising pharmacological potential of these compounds, complementing the mechanistic investigations presented herein. We anticipate that the combination of detailed mechanistic insights and bioactivity predictions will offer a comprehensive understanding of the reactivity and functional relevance of nitropyrazole derivatives. Ultimately, this work is expected to contribute to the formulation of general conclusions regarding the elimination behavior of nitropyrazolines and the broader synthetic and biological implications of 1,3-diaryl-5-nitropyrazoles, as highlighted in the title.

## 2. Results and Discussion

### 2.1. Mechanistic Consideration

Our studies began with the analysis of the reaction course for a model system based on 1,3-diphenyl-4-trichloromethyl-5-nitro-2-pyrazoline (**3a**) in a non-polar environment which is benzene. Initial observations indicated that the transformation is mechanistically straightforward. The conversion of the starting molecule (**3a**) into the target nitropyrazole (**4a**) proceeds via a single TS, without the formation of any detectable intermediates or pre-reaction complexes at the early stages of the reaction. This behavior is typical for systems undergoing single-step eliminations, where a single transition state dominates the transformation, a feature also observed in similar heterocyclic eliminations [49,50]. According to ωB97xD/6-31+G(d,p) (PCM) calculations, the progression of the system toward the transition state (TS) is accompanied by an enthalpy increase of approximately 30 kcal/mol (Table 1). The Gibbs free energy of activation closely mirrors this value, indicating that entropy changes during this stage are negligible. These energy profiles suggest a well-defined single-step pathway, with no competing mechanistic channels in this reaction environment.

**Figure 1:**
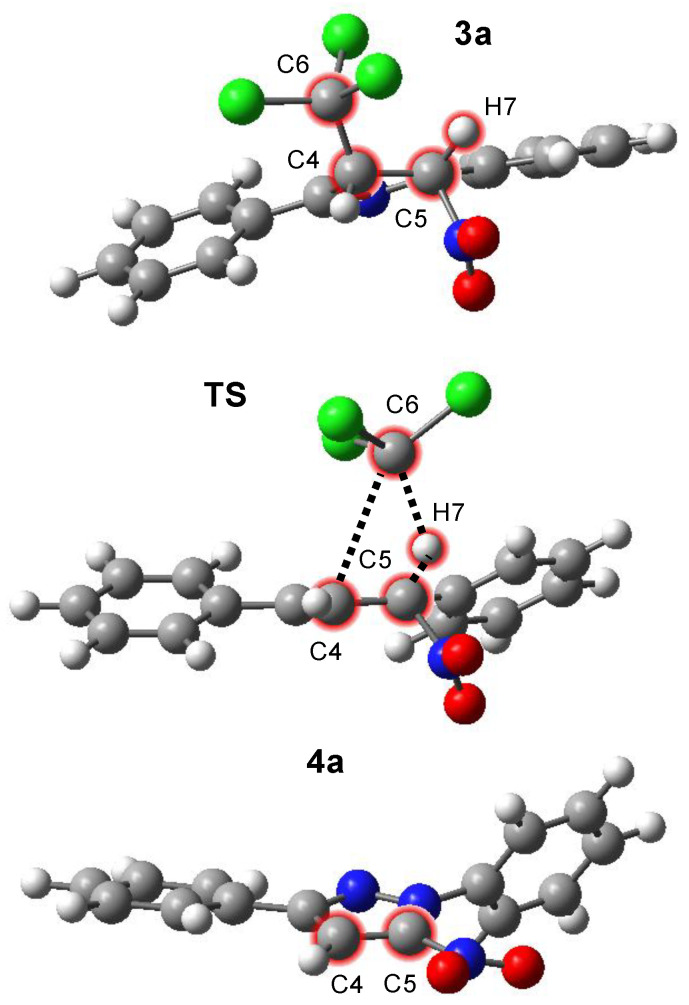
Views of critical structures of the processes of the formation of 1,3-diphenyl-5-nitropyrazoles (**4a**), according to ωB97xD/6-31+G(d,p) calculations in benzene solution (PCM).

The optimized TS exhibits a strained, four-membered cyclic geometry (Figure 1, Table 2). Within this structure, two σ-bonds are broken, namely the C4–C6 bond and the C5–H7 bond. Simultaneously, a new C6–H7 bond is formed, and the C4–C5 bond undergoes a change in character due to *sp^3^*-*sp^2^* rehybridization at the C4 and C5 carbon atoms. This kind of bond reorganization is typical for eliminations involving strained cyclic TSs. The geometrical arrangement highlights the one-step nature of the elimination, where multiple bond changes occur at the same time.

Despite occurring in a single step, the electron density redistribution and bond-breaking events are highly asynchronous. Specifically, the dissociation of the C6 carbon atom occurs substantially faster than the cleavage of the H7 hydrogen atom. These features indicate that, although single-step, the reaction does not follow a classical pericyclic mechanism, but rather a single-step E1-like elimination. IRC calculations further corroborate the mechanistic picture by directly connecting the optimized TS with both the reactant (**3a**) and the product (**4a**). This direct transformation (Wróblewska) is typical for single-step eliminations, where no intermediates are detected along the reaction path, confirming the single-step nature of the transformation [51].

**Figure 2:**
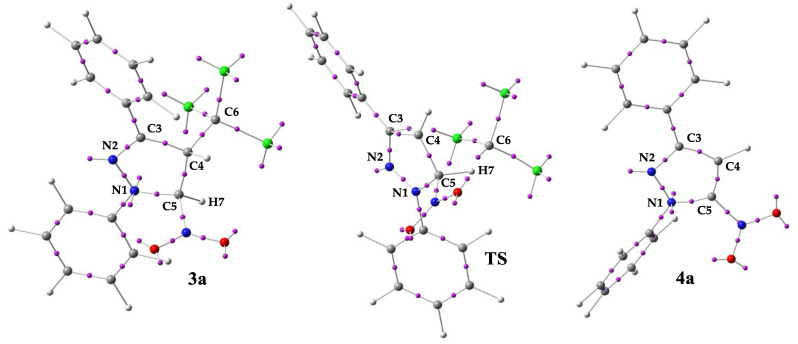
ELF attractor positions (pink spheres) of the core and valence basins with the respective atom numbers, shown for selected critical structures along the reaction pathway leading to the formation of 1,3-diphenyl-5-nitropyrazoles (**4a**).

**Figure 3:**
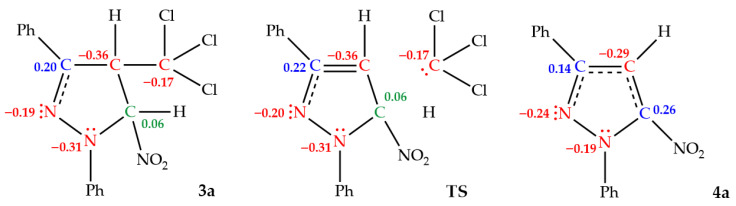
Proposed ELF-based Lewis-like structures with the natural atomic charges for selected critical structures along the reaction pathway leading to the formation of 1,3-diphenyl-5-nitropyrazoles (**4a**) (given an average number of electrons, e). Positive charges are colored blue, negative charges are colored red, while negligible charges are colored green.

To gain deeper insight into the mechanistic nature of the elimination process considered in this study, an Electron Localization Function (ELF) analysis was performed for the model system based on transformation of 1,3-diphenyl-4-trichloromethyl-5-nitro-2-pyrazoline (**3a**) to 1,3-diphenyl-5-nitropyrazole (**4a**). This approach provides a detailed picture of electron density reorganization and allows for a more precise characterization of the bond-breaking and bond-forming events underlying the reaction pathway. The ELF technique itself represents a powerful and widely applicable tool for analyzing electronic structures [41]. By quantifying the probability of finding electrons in the vicinity of a reference electron with the same spin [52], it provides direct insight into the organization of electron density. This makes ELF particularly useful for studying molecular bonding, reactivity, and mechanistic pathways [53,54]. In the case of elimination reactions, ELF analysis allows tracking of electron pair reorganization during bond cleavage and bond formation, which helps to elucidate the nature of the transition state and to distinguish between concerted and stepwise mechanisms [55]. Thus, the ELF attractor positions of the core and valence basins for selected critical structures along the reaction pathway of the transformation of 1,3-diphenyl-4-trichloromethyl-5-nitro-2-pyrazoline (**3a**) into 1,3-diphenyl-5-nitropyrazoles (**4a**) are shown in Figure 2, while the most relevant valence basin populations for these structures are given in Table 3. Based on the ELF analysis, the proposed Lewis-like structures were constructed and are presented in Figure 3, together with the natural atomic charges derived from the NPA.

The ELF analysis of selected critical structures along the reaction pathway leading to the formation of 1,3-diphenyl-5-nitropyrazoles (**4a**) reveals a pronounced electron reorganization throughout the entire course of the elimination (Figure 2). In particular, the ELF results confirm that compound **3a** exhibits an arrangement characteristic of a Δ^2^-pyrazoline ring (Figure 3). For the examined structure **3a**, the most relevant is the presence of disynaptic basin V(N2,C3), which integrates to an electron population of 3.12 e (Table 3). The presence of this basin is associated with a strongly depopulated N2–C3 one-and-a-half-order bond (Figure 3). This effect is attributed to the withdrawal of electron density by the nitrogen atom N2, which hosts a monosynaptic basin V(N2) (Figure 2) integrating to 2.81 e (Table 3), corresponding to approximately three electrons. Two monosynaptic basins, V(N1) and V′(N1), are also located on the second nitrogen atom N1 within the heterocyclic ring (Figure 2). In this case, both basins integrate to a total of 2.34 e (Table 3), which can be associated with the presence of two electrons on the N1 atom.

For the TS structure of the considered transformation, the monosynaptic basins on the N1 atom disappear (Figure 2). On the other hand, the electron population between the two nitrogen atoms, N1 and N2, within the heterocyclic ring increases. In this case, the disynaptic basin V(N1,N2) integrates to 2.99 e (Table 3), which is indicative of a one-and-a-half-order bond (Figure 3). A similar increase in electron population is observed between the carbon atoms C3 and C4, for which two disynaptic basins, V(C3,C4) and V′(C3,C4), are identified (Figure 2). Each of them integrates to 1.80 e (Table 3), which together can be associated with the presence of a double bond between C3 and C4 atoms (Figure 3). Finally, the electron population between the nitrogen atom N2 and the carbon atom C3 decreases to 2.07 e (Table 3), a value typically consistent with the presence of a single bond (Figure 3). In the considered TS, the appearance of a monosynaptic basin V(C6) is also observed on the carbon atom C6 of the departing trichloromethyl group (Figure 2). This basin integrates to an electron population of 2.28 e (Table 2), which can be associated with a lone electron pair.

The ELF analysis of the final product **4a** qualitatively resembles that of pyrazoline **3a** in terms of the topology of electron localization basins. In particular, two monosynaptic basins, V(N1) and V′(N1), are again present on the nitrogen atom N1 (Figure 2). These basins integrate to a total electron population of 1.75 e (Table 3), which is lower than that observed for the corresponding N1 atom in pyrazoline **3a**. Conversely, in pyrazole **4a**, a noticeable increase in the electron population of the monosynaptic basin V(N2) is observed on the adjacent nitrogen atom N2, reaching 3.06 e compared to its counterpart in pyrazoline **3a** (Table 3). This increase effectively compensates for the charge distribution within this region of the ring. Additionally, the electron population in the disynaptic basin V(N1,N2) increases, integrating to 1.62 e (Table 3), yet still indicating a single bond character between these atoms (Figure 3). A similar situation is observed between the nitrogen atom N1 and the carbon atom C5. The disynaptic basin V(N1,C5) integrates to 2.20 e (Table 3), suggesting a slightly overpopulated single bond (Figure 3).

The analysis of the electron populations between the remaining atoms in the heterocyclic ring of pyrazole **4a** indicates that the ring acquires an aromatic character. In particular, the populations of the disynaptic basins V(N2,C3), V(C3,C4), and V(C4,C5), amounting to 2.51 e, 2.91 e, and 3.31 e, respectively (Table 3), suggest the presence of one-and-a-half-order bonds between these atoms (Figure 3). Among them, the C4–C5 interaction, corresponding to the bond formed upon elimination, exhibits the highest electron population (Table 3), being closest in character to a double bond.

It should also be emphasized that all attempts to locate or optimize critical structures corresponding to alternative, stepwise mechanisms were unsuccessful. Consequently, the mechanism presented here should be regarded as the only plausible pathway for the title reaction.

In the second step, we examined the influence of solvent polarity on the reaction course. Our analysis revealed that increasing the polarity of the reaction medium leads to a noticeable reduction in the activation barrier, sometimes by several kilocalories per mole. This finding underscores the polar nature of the analyzed asynchronous process, indicating that the transition state is stabilized to some extent by polar environments [55,56]. In addition to the decrease in activation enthalpy (Table 1), we observed that the key C4–C6 interatomic distance becomes noticeably longer in more polar solvents (Table 2). This elongation further enhances the asynchronicity of the transition state, reflecting a greater temporal separation between bond-breaking and bond-forming events. Despite this subtle modulation, the effect is not sufficient to promote an alternative elimination mechanism; the reaction continues to proceed via the single-step, highly asynchronous pathway observed in non-polar solvents. Finally, IRC calculations confirmed that the overall reaction pathway remains essentially unchanged regardless of solvent polarity. The reactant, transition state, and product are connected in the same manner as in the benzene solution, demonstrating that solvent effects primarily influence the energy profile and the degree of asynchronicity rather than the fundamental mechanism itself. These results collectively indicate that while polar solvents can slightly modulate the kinetics and electronic distribution of the reaction, they do not alter the intrinsic single-step character of the CHCl_3_-elimination.

A similar analysis was carried out to evaluate the influence of substituents on the reaction course. For this part of the study, we selected four substituted molecular models, incorporating either strong electron-donating groups (EDG_S_, e.g., amino groups) or strong electron-withdrawing groups (EWG_S_, e.g., nitro groups), as defined by their respective Hammett constants. Our results revealed that the presence of EWG substituents on the phenyl rings leads to both quantitative and qualitative changes in the reaction. Quantitatively, the activation barrier is increased, making the reaction slower compared to the unsubstituted or EDG-substituted analogs (Table 1). Qualitatively, the asynchronicity of the transition state decreases, indicating that the bond-breaking and bond-forming events become more synchronous under the influence of EWG_S_ (Table 2).

In contrast, molecular models containing EDG substituents participate in the elimination process via a relatively lower activation barrier (Table 1) and a more asynchronous transition state (Table 2). This reflects a faster reaction rate and a greater temporal separation between bond cleavage and bond formation events. Importantly, these substituent-induced changes are subtle; while they modulate the kinetics and electron density redistribution, they are not sufficient to alter the fundamental single-step elimination mechanism. The consistency of these observations was confirmed by IRC calculations, which demonstrated that the optimized transition states remain directly connected to their respective reactants and products, regardless of the nature of the substituents.

Taken together, these findings indicate that the single-step, highly asynchronous mechanism of chloroform elimination can be considered general across a range of molecular segments and solvents. The substituents influence the reaction quantitatively (activation barriers, bond distances) and slightly qualitatively (degree of asynchronicity), but they do not generate alternative elimination pathways. This reinforces the robustness and predictive applicability of the proposed mechanism for 1,3-diaryl-5-nitropyrazoles.

### 2.2. In Silico Evaluation of Biological Potential

Pyrazoles and their derivatives constitute an important class of heterocycles widely recognized for their broad spectrum of biological and pharmacological activities [57,58]. Their structural flexibility and ability to participate in hydrogen bonding make them privileged scaffolds in drug discovery, comparable to other nitrogen containing heterocycles such as imidazoles, triazoles, and isoxazoles [59,60]. Numerous studies emphasize that substitution patterns on the pyrazole core strongly influence activity profiles, enabling fine tuning of lipophilicity, solubility, and metabolic stability [61,62]. In particular, the introduction of a nitro group into the heteroaromatic framework significantly modifies electronic properties and often enhances antimicrobial, anticancer, or anti-inflammatory activity [6,7]. These features make this class of compounds attractive candidates for further evaluation of their application potential.

In light of the above, we performed a preliminary in silico evaluation of the potential biological applications of 1,3-diaryl-5-nitropyrazoles (**4a**–**e**). The basic physicochemical properties, along with selected pharmacokinetic parameters, were calculated using the SwissADME web tool [63]. The resulting data were then assessed according to the established criteria of Lipinski et al. [64], Ghose et al. [65], Veber et al. [66], Egan et al. [67], and Muegge et al. [68]. Finally, the potential biological activities and possible application pathways for compounds **4a**–**e** were predicted using the PASS software [69].

#### 2.2.1. ADME In Silico Evaluation of Pharmacokinetic Properties

**Figure 4:**
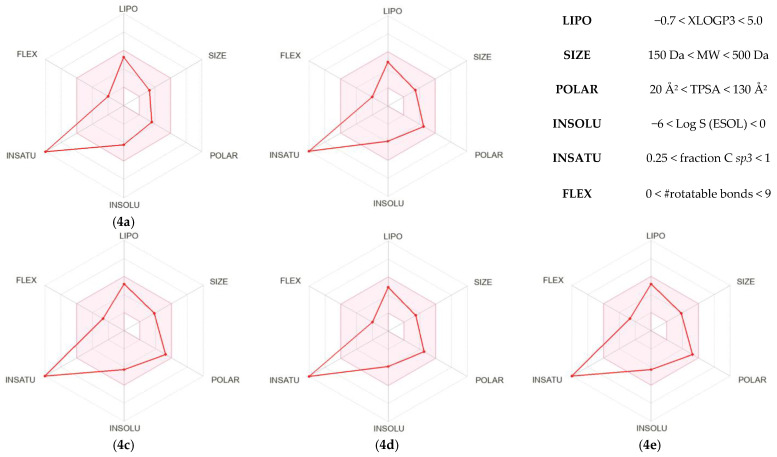
SwissADME [63] bioavailability radars for 1,3-diaryl-5-nitropyrazoles (**4a**–**e**) show the drug-likeness profile, with the pink area defining the optimal range for lipophilicity, size, polarity, solubility, saturation, and flexibility.

ADME investigations are essential in the early phases of drug development. Conducting experimental studies to determine pharmacokinetic parameters can be both time-consuming and resource-intensive. Consequently, computational approaches are increasingly applied to predict ADME properties and optimize research efficiency. In this study key physicochemical attributes including lipophilicity, solubility, pharmacokinetic behavior and medicinal chemistry suitability of the 1,3-diaryl-5-nitropyrazoles (**4a**–**e**) were assessed. The results of these analyses for compounds **4a**–**e** are compiled in Table 4 while their corresponding bioavailability radars are illustrated in Figure 4. Furthermore, the drug-likeness of these molecules was evaluated using the freely available SwissADME platform [70,71] which offers reliable in silico predictions to aid the design of potential drug candidates [72,73].

Analysis of the physicochemical properties and predicted ADME parameters presented in Table 3 indicates that all tested 1,3-diaryl-5-nitropyrazoles (**4a**–**e**) are promising candidates for drug development according to all applied filters. Specifically, all compounds **4a**–**e** meet the criteria regarding molecular weight, number of rotatable bonds, hydrogen bond donor–acceptor balance, and exhibit satisfactory Topological Polar Surface Area (TPSA) values (Table 4). TPSA reflects the total surface area of polar atoms such as nitrogen, oxygen, and sulfur and is useful for predicting membrane permeability and drug bioavailability. Generally, TPSA values below 140 Å^2^ are associated with good oral bioavailability, while values below 90 Å^2^ are favorable for blood–brain barrier penetration [74,75]. For all considered molecules **4a**–**e**, these values fall within the range of 60–90 Å^2^ (Table 4), supporting favorable membrane permeability and potential oral bioavailability.

Molar refractivity (MR) also constitutes a key parameter. It reflects both the polarizability and the size of a molecule, which are critical for its interactions with biological targets. According to the *Ghose* filter [65], optimal MR values range from 40 to 130 cm^3^/mol. This range indicates molecular dimensions and flexibility that favor strong and specific binding to protein active sites. Values below this range may correspond to small, poorly polarizable molecules. Such molecules may form weak interactions and be rapidly eliminated, reducing pharmacological effectiveness. Conversely, excessively high MR values suggest large, rigid molecules with limited flexibility. These molecules may have impaired membrane permeability, decreased solubility, and increased nonspecific interactions, affecting bioavailability and safety [76]. The 1,3-diaryl-5-nitropyrazoles (**4a**–**e**) examined in this study exhibit MR values between 77 and 87 cm^3^/mol (Table 4). These values are consistent with optimal molecular size and polarizability, supporting favorable interactions with biological targets and promising pharmacokinetic properties.

The analysis of lipophilicity and water solubility shows that all tested 1,3-diaryl-5-nitropyrazoles (**4a**–**e**) have good lipophilic properties and adequate aqueous solubility (Table 4). These physicochemical properties are essential for drug absorption and metabolism [77]. Lipophilicity supports membrane permeability. Water solubility ensures sufficient concentration in bodily fluids. Both properties are necessary for effective gastrointestinal absorption [78]. Lipophilicity also affects metabolic enzyme activity, influencing compound stability and elimination [79,80]. The consensus Log P_o/w_ values [81], representing the average octanol/water partition coefficient [82], fall within the favorable range of 0–3 for all compounds (Table 4). Water solubility, expressed as Log S, provides insight into the ability of compounds to dissolve in bodily fluids. According to the current scale [71], molecules with Log S below −4 are moderately soluble in water. Solubility improves as Log S increases.

According to predictions based on SwissADME software, all considered 1,3-diaryl-5-nitropyrazoles (**4a**–**e**) exhibit good gastrointestinal (GI) absorption (Table 4). High GI absorption is important because it indicates that the compounds can efficiently pass through the intestinal lining and enter systemic circulation, which is essential for oral bioavailability and therapeutic effectiveness [83,84]. Furthermore, based on the evaluated pharmacokinetic parameters presented in Table 3, all tested compounds (**4a**–**e**) show low blood–brain barrier (BBB) permeability. Compounds that readily cross the BBB can exert pharmacological effects in the brain, which is important for drugs targeting the central nervous system. In contrast, low BBB permeability is desirable for drugs that should not affect the brain, as it reduces the risk of central nervous system side effects [85]. Finally, all tested compounds (**4a**–**e**) selectively do not inhibit two of the five evaluated cytochrome P450 isoforms, namely CYP2D6 and CYP3A4, while inhibiting the others (Table 4). This is important because it suggests that these compounds are less likely to cause drug–drug interactions involving the major CYP isoforms responsible for the metabolism of most clinically used drugs.

The presented information are fully consistent with the bioavailability radars. They reveal one important aspect that was not considered previously, which is the degree of unsaturation. For all the considered 1,3-diaryl-5-nitropyrazoles (**4a**–**e**), this value is 100% (Figure 4). This feature is important for the interpretation of future predictive studies of these molecules, but it does not necessarily pose a limitation and can be compatible with favorable pharmacokinetic and biological properties [86].

#### 2.2.2. PASS in Silico Assessment of Biological Activity

Due to the promising biological potential of 1,3-diaryl-5-nitropyrazoles (**4a**–**e**), their activity spectrum was predicted using the PASS tool [48], available through the Way2Drug portal [69]. PASS is a computational method that estimates drug-likeness, mechanisms of action, pharmacological effects, toxicity, and other parameters relevant in early drug discovery [87]. Despite relying only on 2D structural descriptors, it provides rapid and accurate predictions that can guide further in silico and experimental studies [71]. Results are expressed as the probability of activity (Pa) versus inactivity (Pi), with compounds considered active when Pa > Pi [48]. A Pa value above 0.700 indicates high probability of activity and priority for experimental validation, whereas values between 0.500–0.700 suggest moderate probability. Predictions below 0.500 are regarded as low confidence [48]. For all investigated compounds (**4a**–**e**), the predicted biological activities that met the Pa > Pi and Pa > 0.700 thresholds are summarized in Table 5.

The PASS prediction shows that the main biological potential of the studied 1,3-diaryl-5-nitropyrazoles (**4a**–**e**) is associated with their inhibitory activity. The most consistent predictions for all compounds include inhibition of hyponitrite reductase, (R)-6-hydroxynicotine oxidase, acrocylindropepsin, saccharopepsin, and chymosin (Table 5). These targets represent oxidoreductases and proteases of therapeutic interest. Oxidoreductases play a central role in cellular redox homeostasis and metabolic regulation, making them attractive targets in the treatment of oxidative stress-related disorders and infections [88]. Proteases such as pepsins and chymosin are crucial in protein processing and turnover, and their selective inhibition has been widely explored in therapeutic strategies against cancer, inflammatory diseases, and microbial pathogens [89]. The predicted inhibitory activity therefore indicates that the studied pyrazoles (**4a**–**e**) and their analogs may hold relevance for multiple pharmacological applications.

A notable observation is that compounds **4b** and **4d** with an amino group in the ring show no activities with Pa > 0.700, whereas nitro-substituted compounds display clear inhibitory potential. This highlights the key role of the nitro group in enhancing biological activity [90,91]. Moreover, the position of the nitro group in the pyrazole ring does not alter either the predicted biological applications or the probability values. Such consistency is unusual and further underlines the strong and reliable influence of the nitro group on the activity profile of these compounds [16].

## 3. Computational Details

All calculations were carried out with the Gaussian 16 package [92], using the Ares cluster at the CYFRONET regional computing center in Cracow. DFT computations employed the hybrid ωB97X-D functional [93], which accounts for long-range exchange (X) and London-dispersion (D) corrections. The 6-31+G(d,p) basis set [94] was employed, which includes diffuse functions on heavy atoms as well as polarization functions, with d-type functions added for second-row elements and p-type functions for hydrogens. Reaction mechanisms were analyzed within the MEDT framework [95,96,97,98], a widely used approach for studying both organic and inorganic processes, particularly cycloadditions, elimination, and intramolecular rearrangements [99,100]. We compared the data obtained in this way with the results of calculations using more advanced basis sets. It turned out that the differences were very small, and therefore the level of theory used was completely sufficient to solve the problem in question (Table 6).

Geometry optimizations of all critical structures were performed under standard conditions in the gas phase at 298 K and 1 atm. Vibrational frequency analyses confirmed that the reactants and products correspond to true minima with all positive Hessian eigenvalues [101]. Solvent effects were taken into account through full reoptimization of the gas-phase geometries at the same level of theory within the polarizable continuum model (PCM) [102] using the self-consistent reaction field (SCRF) formalism [103,104].

Transition states were optimized using the Berny algorithm [105,106] and verified by vibrational frequency analysis, which showed the presence of a single imaginary frequency. The intrinsic reaction coordinate (IRC) method [107] with the second-order González–Schlegel integration scheme [108,109] was employed to confirm the correct connection between each transition state and the corresponding minima. The electronic structure of selected transition state were characterized by the Electron Localization Function (ELF) [52] and the Natural Population Analysis (NPA) [110,111]. The ELF studies were performed with TopMod 09 [112] software.

The physicochemical descriptors and pharmacokinetic properties of the studied compounds were calculated using the SwissADME online server [63]. This platform enabled the evaluation of absorption, distribution, metabolism, and excretion parameters, providing a comprehensive picture of the molecular behavior in a biological context. To further assess the drug-likeness of the designed structures, several well-established predictive models were employed, including the rules proposed by Lipinski et al. [64], Ghose et al. [65], Veber et al. [66], Egan et al. [67], and Muegge et al. [68]. These filters collectively allowed for a systematic examination of the molecular features that influence oral bioavailability and therapeutic potential. In addition, the Prediction of Activity Spectra for Substances online tool [69] was utilized to estimate the probability of specific biological activities. This approach, based on structure–activity relationship (SAR) modeling, provided valuable insight into the potential pharmacological profiles of the investigated pyrazole derivatives, complementing the ADME-based evaluation.

Molecular geometries of all structures were visualized with the GaussView 6.0 software [113], while the ELF localization domains were generated using ParaView 5.9.1 [114] with an isovalue set to 0.75 a.u. The bioavailability radars were adopted from the SwissADME online server [63].

## 4. Conclusions

Our comprehensive wB97X-D/6-31+G(d,p) (PCM) quantum-chemical study provides a complete picture of the molecular mechanism underlying the formation of 1,3-diaryl-5-nitropyrazoles from trichloromethylated diarylnitropyrazolines. The obtained results show that the conversion of pyrazolines into the target 1,3-diaryl-5-nitropyrazoles proceeds via a single-step mechanism following a chloroform elimination pathway. The analysis of solvent and substituent effects confirms that the proposed reaction pathway can be considered general, as it is independent of both the nature of the substituents on the phenyl rings and the polarity of the reaction medium. According to the current nomenclature, the described mechanism should be classified as a pseudocyclic, non-concerted, E1-like elimination. Full exploration of details of the electron density distribution within selected critical structures reveals a pronounced electron density reorganization accompanying the studied transformations. The progressive depopulation and formation of selected disynaptic basins reflect the redistribution of electron density within the reacting system, closely associated with the evolution of bond character and the development of conjugated π-structures. The observed changes in monosynaptic and disynaptic basin populations consistently indicate a shift toward enhanced electron delocalization, ultimately leading to the stabilization of the heterocyclic framework. Such features are characteristic of the formation of aromatic or quasi-aromatic systems in the final stages of the reactions.

The combined PASS and ADME analyses indicate that the studied 1,3-diaryl-5-nitropyrazoles possess favorable physicochemical and pharmacokinetic profiles, consistent with drug-like molecules. All compounds meet the general criteria of molecular stability, solubility, and permeability, showing good gastrointestinal absorption and low blood–brain barrier penetration. The predicted bioavailability and limited interaction with key cytochrome P450 isoforms suggest a low risk of metabolic interference. According to PASS predictions, the compounds display a notable inhibitory potential, particularly toward oxidoreductases and proteases, which may be relevant for therapeutic applications in oxidative stress-related and inflammatory conditions. The results also underline the essential role of the nitro group in enhancing biological activity, confirming its key influence on the pharmacological behavior of this class of compounds.

## Figures and Tables

**Table 1:** Energetical parameters for processes of the formation of 1,3-diaryl-5-nitropyrazoles (**4a**–**e**), according to ωB97xD/6-31+G(d,p) (PCM) calculations (ΔH, ΔG are in kcal/mol; ΔS are in cal/mol·K). Dielectric constants are abbreviated as ε.

Solvent	Substrate	Transition	ΔH	ΔS	ΔG
Benzene	**3a**	**3a** → **TS**	34.7	8.2	32.2
(ε = 2.2706)		**3a** → **4a**	−40.4	45.2	−53.9
Acetone	**3a**	**3a** → **TS**	30.6	9.0	27.9
(ε = 20.493)		**3a** → **4a**	−41.1	45.8	−54.7
Nitromethane	**3a**	**3a** → **TS**	30.3	−0.9	30.6
(ε = 36.562)		**3a** → **4a**	−41.1	35.7	−51.7
Benzene	**3b**	**3b** → **TS**	33.5	9.4	30.7
		**3b** → **4b**	−39.8	47.1	−53.9
Benzene	**3c**	**3c** → **TS**	36.1	8.7	33.5
		**3c** → **4c**	−41.0	45.5	−54.6
Benzene	**3d**	**3d** → **TS**	28.2	8.2	25.7
		**3d** → **4d**	−42.6	47.4	−56.8
Benzene	**3e**	**3e** → **TS**	40.5	11.4	37.1
		**3e** → **4e**	−37.4	48.6	−51.9

**Table 2:** Key interatomic distances in critical structures of the processes of the formation of 1,3-diaryl-5-nitropyrazoles (**4a**–**e**), according to ωB97xD/6-31+G(d,p) (PCM) calculations. Dielectric constants are abbreviated as ε.

Solvent	Reaction	Structure	Interatomic Distances [Å]			
			**C4**–**C6**	**C6**–**H7**	**H7**–**C5**	**C4**–**C5**
Benzene	**3a** → **4a**	**3a**	1.552	2.666	1.090	1.539
(ε = 2.2706)		**TS**	3.339	2.155	1.106	1.482
		**4a**				1.375
Acetone	**3a** → **4a**	**3a**	1.552	2.674	1.089	1.538
(ε = 20.493)		**TS**	3.398	2.261	1.101	1.481
		**4a**				1.375
Nitromethane	**3a** → **4a**	**3a**	1.552	2.675	1.089	1.538
(ε = 36.562)		**TS**	3.402	2.270	1.101	1.481
		**4a**				1.375
Benzene	**3b** → **4b**	**3b**	1.552	2.664	1.089	1.539
		**TS**	3.355	2.173	1.104	1.480
		**4b**				1.374
Benzene	**3c** → **4c**	**3c**	1.553	2.661	1.089	1.540
		**TS**	3.295	2.136	1.108	1.481
		**4c**				1.374
Benzene	**3d** → **4d**	**3d**	1.552	2.670	1.090	1.539
		**TS**	3.452	2.195	1.101	1.483
		**4d**				1.375
Benzene	**3e** → **4e**	**3e**	1.553	2.664	1.090	1.536
		**TS**	3.327	2.129	1.109	1.480
		**4e**				1.372

**Table 3:** Most significant ELF valence basin populations for selected critical structures along the reaction pathway leading to the formation of 1,3-diphenyl-5-nitropyrazoles (**4a**).

	Valence Basin Populations, N [e]		
**ELF Basins**	**3a**	**TS**	**4a**
**V(N1)**	1.01		0.87
**V′(N1)**	1.33		0.88
**V(N1,N2)**	1.44	2.99	1.62
**V(N2)**	2.81	2.67	3.06
**V(N2,C3)**	3.12	2.07	2.55
**V(C3,C4)**	2.02	1.80	2.91
**V′(C3,C4)**		1.80	
**V(C4,C5)**	1.96	2.07	3.39
**V(N1,C5)**	1.78	2.00	2.20
**V(C4,C6)**	2.02		
**V(C6)**		2.28	

**Table 4:** Drug-likeness parameters for 1,3-diaryl-5-nitropyrazoles (**4a**–**e**) were calculated using the SwissADME [63].

		4a	4b	4c	4d	4e
*Physchem. properties*	**MW [g/mol]**	265.27	280.28	310.26	280.28	310.26
	**#heavy atoms**	20	21	23	21	23
	**#arom. heavy atoms**	17	17	17	17	17
	**#rotatable bonds**	3	3	4	3	4
	**#H-bond acceptors**	3	3	5	3	5
	**#H-bond donors**	0	1	0	1	0
	**MR**	77.82	82.23	86.64	82.23	86.64
	**TPSA [Å^2^]**	63.64	89.66	109.46	89.66	109.46
*Lipophilicity*	**Log P_o/w_ (iLOGP)**	2.32	1.93	2.03	1.89	2.02
	**Log P_o/w_ (XLOGP3)**	3.72	3.03	3.54	3.03	3.54
	**Log P_o/w_ (WLOGP)**	3.97	3.56	4.40	3.56	4.40
	**Log P_o/w_ (MLOGP)**	3.63	2.27	2.64	2.27	2.64
	**Log P_o/w_ (SILICOS-IT)**	1.21	0.48	−0.58	0.48	−0.58
	**Consensus Log P_o/w_**	2.97	2.26	2.41	2.25	2.41
*Water solubility*	**Log S (ESOL)**	−4.26	−3.89	−4.28	−3.89	−4.28
	**solubility [mg/mL]**	0.0146	0.0363	0.0164	0.0363	0.0164
	**Log S (Ali)**	−4.75	−4.58	−5.52	−4.58	−5.52
	**solubility [mg/mL]**	0.0047	0.0074	0.0093	0.0074	0.0093
	**Log S (SILICOS-IT)**	−5.30	−4.94	−5.15	−4.94	−5.15
	**solubility [mg/mL]**	0.0013	0.0032	0.0021	0.0032	0.0021
Pharmacokinetics *	**CYP1A2 inhibitor**	Yes	Yes	Yes	Yes	Yes
	**CYP2C19 inhibitor**	Yes	Yes	Yes	Yes	Yes
	**CYP2C9 inhibitor**	Yes	Yes	Yes	Yes	Yes
	**CYP2D6 inhibitor**	No	No	No	No	No
	**CYP3A4 inhibitor**	No	No	No	No	No
	**IG absorption**	Yes	Yes	Yes	Yes	Yes
	**BBB permeant**	Yes	No	No	No	No
*Druglikeness*	**Lipinski et al.** [64] **(Pfizer)**	✔	✔	✔	✔	✔
	**Ghose et al.** [65] **(Amgen)**	✔	✔	✔	✔	✔
	**Veber et al.** [66] **(GSK)**	✔	✔	✔	✔	✔
	**Egan et al.** [67] **(Pharmacia)**	✔	✔	✔	✔	✔
	**Muegge et al.** [68] **(Bayer)**	✔	✔	✔	✔	✔

* Explanation: Yes—The compound is **predicted to possess** the property in question; No—The compound is **predicted not to possess** the property in question.

**Table 5:** The PASS prediction [69] for 1,3-diaryl-5-nitropyrazoles (**4a**–**e**) indicates their main potential activities, with results expressed as the probability of activity (Pa) values above 0.700.

	4a	4c	4e
5-O-(4-coumaroyl)-D-quinate 3′-monooxygenase inhibitor	0.751	-	-
Hyponitrite reductase inhibitor	0.741	0.758	0.758
(R)-6-hydroxynicotine oxidase inhibitor	0.737	0.747	0.747
Acrocylindropepsin inhibitor	0.748	0.808	0.808
Saccharopepsin inhibitor	0.748	0.808	0.808
Chymosin inhibitor	0.748	0.808	0.808
Glycosylphosphatidylinositol phospholipase D inhibitor	0.728	-	-
Phospholipid-translocating ATPase inhibitor	0.708	-	-
Arylacetonitrilase inhibitor	0.704	0.712	0.712
(R)-6-hydroxynicotine oxidase inhibitor	-	0.747	0.747
Fusarinine-C ornithinesterase inhibitor	-	0.741	0.741
Ubiquinol-cytochrome-c reductase inhibitor	-	0.765	0.765
Bisphosphoglycerate phosphatase inhibitor	-	0.728	0.728
Phospholipid-translocating ATPase inhibitor	-	0.716	0.716

**Table 6:** Key interatomic distances in TS of the processes of the formation of 1,3-phenyl-5-nitropyrazole (**4a**) in the benzene solution calculated at different levels of theory.

	Interatomic Distances [Å]			
Level of Theory	C4–C6	C6–H7	H7–C5	C4–C5
ωB97X-D/6-31+G(d,p)	3.339	2.155	1.106	1.482
ωB97X-D/6-311G(d)	3.333	2.168	1.103	1.479
ωB97X-D/6-311G(d,p)	3.320	2.168	1.104	1.478
ωB97X-D/6-311+G(d)	3.344	2.182	1.102	1.479
ωB97X-D/6-311+G(d,p)	3.337	2.180	1.104	1.479

## Data Availability

The data presented in this study are available on request from the corresponding author.

## Abbreviations and Symbols

The following abbreviations and symbols are used in Table 4 and correspond to the physicochemical and pharmacokinetic parameters predicted using the SwissADME tool:

MW	Molecular Weight
#heavy atoms	Number of Heavy Atoms
#arom. heavy atoms	Number of Aromatic Heavy Atoms
#rotatable bonds	Number of Rotatable Bonds
#H-bond acceptors	Number of Hydrogen Bond Acceptors
#H-bond donors	Number of Hydrogen Bond Donors
MR	Molecular Refractivity
TPSA	Topological Polar Surface Area
Log P_o/w_ (iLOGP)	Octanol/Water Partition Coefficient (iLOGP method [115])
Log P_o/w_ (XLOGP3)	Octanol/Water Partition Coefficient (XLOGP3 method [116])
Log P_o/w_ (WLOGP)	Octanol/Water Partition Coefficient (WLOGP method [117])
Log P_o/w_ (MLOGP)	Octanol/Water Partition Coefficient (MLOGP method [118])
Log P_o/w_ (SILICOS-IT)	Octanol/Water Partition Coefficient (SILICOS-IT method [119])
Consensus Log P_o/w_	Consensus Octanol/Water Partition Coefficient
Log S (ESOL)	Predicted Aqueous Solubility (ESOL method [77])
Log S (Ali)	Predicted Aqueous Solubility (Ali method [120])
Log S (SILICOS-IT)	Predicted Aqueous Solubility (SILICOS-IT method [121])
IG absorption	Gastrointestinal Absorption
BBB permeant	Blood–Brain Barrier Permeability
